# Progress of amino acid nutrition for diet protein reduction in poultry

**DOI:** 10.1186/s40104-021-00568-0

**Published:** 2021-04-05

**Authors:** M. T. Kidd, C. W. Maynard, G. J. Mullenix

**Affiliations:** Center of Excellence for Poultry Science, Division of Agriculture, University of Arkansas System, Fayetteville, AR 72701 USA

**Keywords:** Amino acid, Broiler chicken, Glutamine, Histidine, Low protein, Threonine, Valine

## Abstract

There is growing interest among nutritionists in feeding reduced protein diets to broiler chickens. Although nearly a century of research has been conducted providing biochemical insights on the impact of reduced protein diets for broilers, practical limitation still exists. The present review was written to provide insights on further reducing dietary protein in broilers. To construct this review, eighty-nine peer reviewed manuscripts in the area of amino acid nutrition in poultry were critiqued. Hence, nutritional research areas of low protein diets, threonine, glycine, valine, isoleucine, leucine, phenylalanine, histidine, and glutamine have been assessed and combined in this text, thus providing concepts into reduced protein diets for broilers. In addition, linkages between the cited work and least cost formation ingredient and nutrient matrix considerations are provided. In conclusion, practical applications in feeding reduced protein diets to broilers are advancing, but more work is warranted.

## Background

Investigations in reducing dietary crude protein (CP) in chickens began in the early 1940’s, which commenced nearly a century of exploration in this area of poultry nutrition research. Advances have been made, but many of the hurdles identified in the 1940’s vex poultry nutrition researchers today. Most notably, in the early 1940’s research was carried out in chickens fed various diets substituted with ingredients differing in protein and energy content, and it was established that diets with increased protein resulted in chickens having over two fold lower body fat content [1]. In addition to the former effect on carcass composition, low CP-amino acid fortified diet research in the early 1940’s failed to produce birds with equal performance to higher CP fed control birds that consumed less feed grade amino acids [2]. Decades thereafter ensuing industrial growth of broiler production, low CP-amino acid research continued, but it was widely accepted in practice that the dietary inclusion of amino acids beyond the sulfur containing amino acids and lysine would not economically sustain production in practice [3–5]. Towards the end of the twentieth century, adoption of the third feed grade amino acid, i.e., *L*-threonine (Thr), was aided by advances in ideal protein formulation tools and the expression of amino acids in dietary formulation on a digestible basis [6]. At present, *L*-valine (Val), the fourth limiting amino in most broiler diets devoid of meat meals, is being used in practice. Further, the approach of U.S. nutritionists on the use of feed grade amino acids in least cost poultry diets from 1950 to present has been reviewed [7] and is summarized in Table 1. Indeed, formulation adoption of a synthetic methionine source with crystalline lysine, threonine, valine, isoleucine, and arginine can allow for over a 50% reduction in soybean meal and over a 20% reduction in crude protein in a broiler grower diet based on corn, soybean meal, meat and bone meal, and distillers grains.

**Table 1:** Least cost formulation considerations that occurred from 1950 to present allowing for a reduction in CP in poultry diets.

Item	Characteristic
Ingredient matrix	
Removal of feed grade amino acid maximum	The maximum constraint was used to limit the amount of feed grade amino acid entering formulation which prevented deficiencies of other amino acids whereby minimums were unknown, but did so at a cost, with excesses. Its removal reduced CP and diet costs, but required knowledge of most amino acid minimums
Addition of digestible amino acid values to ingredients	The addition of digestible amino acid values for ingredients and use of digestible amino acid nutrient minimums in formation, rather than total levels, allowed for better precision formation
Increased ingredient analyses	More near infrared spectrometer assays were used to compare calculated to predicted analyzed values of numerus ingredients
Nutrient matrix	
Removal of CP nutrient minimums	Least cost diets that solved for CP could be used to assure no deficiencies, but the result is diets that contain excess nitrogen and high costs, as opposed to using amino acid minimums
Addition of digestible amino acid minimums	Formulating to digestible amino acids allowed for reduced CP and more feed grade amino acids meeting shadow values

Production of poultry (e.g., primarily broilers) from the Food and Agriculture Organization of the United Nations 2005/2007 estimate to the 2050 projection will more than double from 82 to 181 million tons of poultry products [8]. In order to provide to the future need for poultry consumption, the bulk of the relative increase in poultry production will occur in developing countries with specific reference to those in Sub-Saharan Africa, North Africa/Near East, South Asia, and East Asia [8]. The former regions are more self-sufficient in cereals than that of oilseeds, deeming the need to broaden practical knowledge for feeding poultry low CP-feed grade amino acid fortified diets to satisfy future food security.

Utilization of the ideal protein concept allows for precision feeding of poultry, but a keen understanding of the birds’ dietary lysine need over time is warranted. However, additional amino acids must be continuously assessed as their minimums can vary based on dietary protein status, bird strain, bird environment, and company production objectives to satisfy economics [9]. As such, this review will provide an assessment of key amino acid considerations in the low CP puzzle and future research considerations which have been summarized in Table 2.

**Table 2:** On reduced crude protein diets for broilers: consideration for the linear programming matrices.

Item	Characteristic
Ingredient matrix	
Soybean meal	Improved varieties: amino acid levels and digestibility, carbohydrate fraction adjustments, improved oil content, and ability to utilize full fat beans
Insect meal	Increased industrialized production of insect meal resulting in decreased costs; improved amino acid digestibility
Algae meal	Increased industrialized production of algae meal resulting in decreased costs; transition from salt water to fresh water systems with reduced climate impact
Single-cell protein meal	Increased industrialized production of single-cell protein sources resulting in decreased costs; improved amino acid digestibility
*L*-valine	Free base *L*-valine is being produced commercially and used in formulation. Its inclusion allows for reduced diet costs, decreased nitrogen excretion, and a mechanism to feed balanced branched-chain amino acids
*L*-glutamine and betaine	Research is necessary to assess if *L*-glutamine and/or betaine can aid intestinal integrity in birds fed low CP diets
Feed-grade amino acids	Increased manufacturing of essential (e.g., beyond *L*-valine) and non-essential amino acids at low cost
Feed-grade amino acid peptides	Increased manufacturing of di- and tri-peptides with good digestibility at low costs
Feed-grade enzymes	Availability of feed-grade enzymes with affordable pricing and consistent efficacy have given nutritionists a tool to improve ingredient quality
Increasing feed phases	Adding diet phases allows for closer meeting the birds’ need and minimizing excesses
Sex separate feeding	As female broilers require an order of 10 to 15% less amino acid levels, separate or separate phased diets per sex can increase nitrogen efficiency
Nutrient matrix	
Protein level	Less dependence on diet protein and more on amino acid supply by formulating to non-essential amino acids
Protein expression	Can converting crude protein to true protein aid in predicting a critical amino acid supply?
Digestive dynamics	As CP decreases, more cereals enter diets with less dietary oil sources, and it is known that feed-grade amino acids interact with starch regarding digestion. Nutritionists should create ratios to limit rapid starch in low CP diets
Ideal protein for digestible amino acids	Robust Lys needs must be assessed as modern broiler genetics continue to be improved and express dietary needs on a digestible basis
Threonine	Threonine must continue to be assessed as antibiotic growth promoters are removed from diets, as well as the balance regarding glycine plus serine
Branched-chain amino acids	Assess limiting needs singularly and together in practical low CP diets
Phenylalanine + tyrosine	Assess limiting needs in low CP diets in and relation to the branched-chain amino acids
Histidine	Assess needs in low CP diets for broilers using a pre-experimental adjustment period
Electrolyte balance	Reduced protein results in less soybean meal and monitoring electrolyte balance from the potassium reduction is critical
Non-essential amino acid levels	As protein continues to be reduced, using nutrient minimums for non-essential amino acids will be required to maintain a nitrogen pool

## Recent low CP research

The increasing interest to lower dietary CP has led to more research defining interrelationships of nutrient digestion and absorption in reduced CP fed broilers. Following the ideal protein concept, a moderate reduction in CP, 20–30 g/kg, maintained performance and processing yields [10, 11]. Lowering CP more than 30 g/kg has been shown to inhibit performance and increase adipose fat deposition [10–12]. Reducing CP in a standard corn/soybean meal diet will lower soybean meal while increasing the proportion of corn, or other cereals, while decreasing feed-grade oils. This results in less intact protein-bound amino acids, increased inclusion of crystalline unbound amino acids and increased starch in the diet.

It was previously determined that intestinal uptakes of glucose and amino acids interrelationships are considered pivotal to broiler performance [13], and the process of starch degradation into glucose in broilers has been described [14]. Further, extent, rate, and site of nutrient digestion should be considered jointly to appropriately balance amino acids and glucose at sites of protein synthesis [15]. Starch and protein digestive dynamics (Table 2) are described as three-tiered process: digestion of starch and protein, absorption of glucose and amino acids from gut lumen, and the transition of glucose and amino acids across enterocytes to enter portal circulation [16]. Starch is more rapidly taken up than that of protein in broilers fed a sorghum-based diet [17]. Starch source plays an important role in its digestive rate and one important focus of digestive dynamics is that starch is digested too rapidly and protein too slowly [18]. Diets with decreased CP and increased starch flood the small intestine with glucose and compete with amino acids for absorption through their respective sodium dependent pathways [19]. Further, starch has a higher ileal digestibility coefficient than protein and amino acids [17, 20], pointing to the need for further research on digestive dynamics in low protein-crystalline amino acid-based diets [21].

Amino acid digestibility in reduced CP diets has been a recent focus. A reduction of 45 g/kg (210 and 165 g/kg) CP effects on broilers raised under tropical conditions significantly increased (0.790 vs. 0.744) average ileal amino acid digestibility coefficients by 6.18% [22]. Reducing CP by 45 g/kg and 30 g/kg increased ileal digestibilities of 16 amino acids by 5.82% and 9.10%, respectively [11, 23]. These fluctuations of ileal digestibility coefficients are credited to a reduction of endogenous amino acid flows in each respective diet. Pepsin, trypsin and remaining endogenous proteolytic enzymes, thus mucin as a large source of endogenous amino acids, are attenuated in reduced CP diets with less intact proteins [24]. The reduced endogenous amino acid flow, and the view that supplemental amino acids are 100% bioavailable and more rapidly absorbed in the proximal small intestine than protein bound amino acids [25], leads to the assumption that less amino acids are being supplied for processes that support gut health. Certain health promoting bacterial groups, that depend on amino acids, are known to stimulate production of mucin and catalyze the proliferation of epithelial cells [26, 27]. Maintaining the optimum function of the epithelial wall in the small intestine is important in controlling permeability, thus reducing incidences of leaky gut. Epithelial cells covering the intestinal villi are linked together through complex proteins; adherens junctions, desmosomes and tight junctions. Tight junctions facilitate paracellular permeability which has a role in absorbing nutrients and preventing entry of microbes and toxins into the body [28]. Increased intestinal permeability can have negative effects on health, bacterial and toxin translocation, lameness and economic losses from impaired performance [29]. Recent research has shown a significant increase in intestinal permeability in birds fed a low protein diet (170/150 g/kg of CP) fortified with all essential amino acids compared to a high protein diet (220/210 g/kg of CP) that exceeded recommendations by 10% [28]. There were no differences of intestinal permeability between a standard protein diet (202/190 g/kg) CP and the reduced protein diet. Reducing CP without the addition of essential amino acids exacerbates the effect of aflatoxicosis on intestinal permeability, which was improved with a 10% increase in CP [30]. These trials display that elevated gut permeability can be observed in birds fed inadequate levels of bound or purified amino acids, and that further research is needed to determine the role of individual amino acid concentrations on leaky gut. The former points to the need to feed the bird for gut integrity when low CP diets are offered, which is primarily highlighted in a subsequent section of this review on glutamine (Gln) (Table 2).

Excessive amino acids (nitrogen) undergo catabolism, presumably from the degradation of imbalanced amino acids, and can accumulate ammonia [31]. Reducing CP 30 g/kg (220 g/kg to 190 g/kg) increased plasma ammonia by 59.4% (7.27 vs. 4.56 mg/dL) and negatively impacted performance [32]. Ammonia detoxification occurs through its reaction with glutamate to form Gln. Glutamine enters the Krebs cycle which produces uric acid and nitrogen waste [33]. Glycine (Gly), serine (Ser), and glutamic acid are also needed for uric acid production, and the former can be limiting in low CP diets. It has been suggested that a minimum Gly + Ser value should be set between 2% to 2.5% [7]. An imbalance of amino acids resulting in inadequate ammonia detoxification could result in ammonia toxicity and reduced performance [31]. Moreover, bird performance could be further reduced due to increased litter nitrogen resulting in footpad lesions. For example, Ross 308 broilers fed reduced CP at 22 g/kg to 23 g/kg had reduced litter nitrogen and footpad lesions, with no adverse effects on performance or yields [34].

## Threonine and glycine linkages

With specific reference to feed, consumer preferences that have been implemented into poultry practice at a rapid rate represent the production of broilers on feed with no antibiotics and no ingredients of animal origin. With reference to sustainable feed solutions, broiler diets will continue to be reduced in CP with improved nitrogen and water balance. Hence, lower CP diets contain less oilseed meals resulting in reduced water intake in broilers. Amino acid nutrition is central in the former preferences and feed solutions, and more specifically, Thr [6] (Table 2).

An assessment of Thr and Gly biochemistry must first consider Gly precursors. Hence, conclusions from early chick trials with Gly considered it to be essential for growth [2]. Serine is a Gly precursor and its dietary abundance was credited for the nonessentiality of Gly in a chick trial [35]. In two trials where low-CP diets were fed to chicks (minimal N-crystalline amino acid diets), Gly and Ser were required for good chick growth, but Ser completely replaced the Gly value on a mole to mole basis [36]. In addition to Ser sparing Gly, Baker and Sugahara [37], conducted two chick trials and demonstrated that choline can partially replace Gly through the pathway of choline to betaine to dimethylglycine to sarcosine to Gly. Hence, variability of research results on Gly needs and Gly by Thr interactions clearly stems from diet variability of Gly precursors.

Similar to that of Ser, Gly precursor knowledge is essential in understanding Gly needs and Thr is the principal Gly precursor in chicks. Thr metabolism with reference to Gly needs has been reviewed [38]. The glucogenic catabolism of Thr occurs through three main pathways through the de novo action of Thr aldolase, Thr dehydrogenase, and Thr dehydratase [38]. Further, both Thr aldolase and Thr dehydrogenase yield Gly to Ser to pyruvate. It must be pointed out that the reverse pathway from Ser to Gly exists. Hence, one-carbon units from formic acid allows for the reversible interconversion of Ser to Gly [39]. In order to determine if the reverse pathway from Gly to Thr existed, Baker et al. [40] conducted a chick trial with end products from Thr aldolase. Chicks fed a Gly rich diet deficient in Thr failed to sustain growth when the diet was fortified with acetaldehyde, indicating the reverse pathway of Gly to Thr is of no biological value in chicks [40]. Therefore, the catabolism of Thr yields pyruvate for energy or Gly to support metabolic processes through the conversion of protein, creatine, Ser, uric acid, guanidino acetic acid, glutathionine, and bile salts [38]. Further, research in chickens has shown that Thr dehydrogenase and Thr aldolase are the most active Thr-degrading enzymes [41].

The previously mentioned commercial trends in poultry production to move towards low or no antimicrobial-feed additive use and all vegetable-based feed ingredients have resulted in an increased awareness of gut inflammation and techniques to mitigate sub-clinical and clinical gut infections. Absorption of nutrients by the upper villus is dependent on mucin, which is partially dependent upon amino acids for integrity. Of the most limiting amino acids for mucin are methionine (Met) and Thr [42]. Methionine for the biosynthesis of cysteine, and Thr for its villi essentiality and the biosynthesis of Gly and Ser [42]. Furthermore, the presence of sub-clinical intestinal insults in poultry can increase the Thr value needed in least cost formulation [43] (Table 2). Similarly, it has been shown that finishing broilers reared on used pine shavings have increased Thr needs compared to those reared on fresh pine shavings [44, 45]. Thus, the Thr value in least cost formulation is dependent upon bird intestinal health and the dietary Gly level. Indeed, the commercial acceptance and use of dietary betaine (Table 2) for osmoregulatory and methyl donation may be interconnected with Thr metabolism. Further, research that assesses Thr and Gly interrelationships in broilers that have mild to moderate enteric stress is warranted, especially in situations where CP varies. Kidd et al. [46] assess CP by Thr interactions in chicks and failed to produce protein efficiency or nitrogen excretion interactions, feed efficiency was improved by CP and Thr interactions (CP linear × Thr linear). Moreover, the former research demonstrated that for each 1% decrease in dietary CP that nitrogen excretion by the bird is decreased approximately 8% [46].

The Gly need in chicks has been shown to be essential early in life and nonessential later in life [47]. Recent work by Kriseldi et al. [48] with starter chicks demonstrated success with low CP diets (2.6% reductions in CP) when the total Gly + Ser to digestible Lys ratio was 190. Similarly, Schutte et al. [49] conducted two trials with Ross chicks (1–14 and 1–21 d) fed corn or wheat and soybean meal-based diets with graduations of Gly and Glu and found responses to Gly, but not Glu. From their work it was concluded that the total Gly + Ser requirement for chicks was between 1.8% and 1.9% [49]. Because total Lys varied from 1.23% to 1.33% of diet, recent work of Kriseldi et al. [48] agrees with Schutte et al. [49]. Work by Heger and Pack [50] indicated that the Gly + Ser need is dependent upon CP. They demonstrated a Gly + Ser chick requirement range of 1.5% to 1.6% at 17% CP and 1.7% to 1.8% at 23% CP, presumably to aid in uric acid synthesis [50]. However, Lys was also increased in the high CP experiment suggesting relevance to a Gly + Ser to Lys ratio [50]. Future work with Gly + Ser, with relevance to Thr needs, should take biosynthetic precursors and gut health into consideration to validate least cost minimum levels and CP reduction potential.

## Branch-chained amino acid interplay

As previously mentioned, *L*-Val is currently being integrated into broiler diets and *L*-isoleucine (Ile) is in the early phases of commercialization, and although diet costs are decreasing potential branched-chain amino acid (BCAA) antagonisms may be increasing. The interconnected nature of the BCAA has been a known phenomenon in poultry since the late 1960's [51], but this interplay has been largely ignored due to a series of articles published in the 1990’s and early 2000’s that indicated that antagonisms among BCAA were of little concern in practical broiler diets [52–54]. Thirty years prior to these experiments, Bray [55] observed similar responses in the practical diets of pullets but postulated that with increases in amino acid production, and their subsequent integration into poultry diets, that these antagonisms could be of particular concern when formulating poultry diets as BCAA excess was reduced.

Overwhelming data suggests that BCAA antagonism is primarily leucine (Leu) mediated, but the exact mechanism involved remains unknown [56–58]. In addition, the antagonism among the BCAA is not limited to Leu excess and can be induced when any of the three are provided at superoptimal levels [56]. These findings have shown that when considering the BCAA, balance among the BCAA is just as important as balancing them in the overall amino acid profile, if not more so. Ousterhout [59] found that feeding diets devoid in Ile and Val resulted in death after 18 and 19 days, respectively, but that when all three of the BCAA were removed in concert, mean survival times were 34 days, effectively doubling their survival time. On the other hand, D’Mello and Lewis [56] found that reductions in body weight gain caused by excess dietary Leu could be corrected by feeding increased levels of Ile and Val. Similarly, Tuttle and Balloun [60] observed that Leu induced reductions in body weight gain in poults and this response could be corrected through additional supplementation of Ile and Val in concert, but not individually.

These observations have led some researchers to theorize that currently implemented BCAA dietary requirements are influenced by inherent BCAA diet levels, leading to an over inflation to counteract the effects of antagonism [61, 62]. Therefore, determining the ratios of BCAA to each other and if these ratios are altered by diet amino acid supply, will be key to formulating to optimal BCAA in reduced CP diets (Table 2). This understanding may not only allow for use of *L*-Val and *L*-Ile in diet formulation, but their overall needed nutrient levels may lower, thus further reducing diet costs.

## Phenylalanine + tyrosine considerations

Sequel to use of synthetic Met, and crystalline Lys, Thr, and Val in broiler diets, several amino acids, formerly thought to be of little practical concern, are poised to impact broiler performance and formulation strategies. Phenylalanine (Phe) has long been taken for granted as it has proven to be one of the least limiting amino acids in broiler diets in the previous decades, but its dietary levels have been observed to be marginally adequate or deficient in some practical diets [63, 64]. Maynard et al. [65] conducted a deletion experiment evaluating the 4th limiting amino acid in corn-soybean meal poultry diets and observed a negative effect from Phe deletion on carcass parts weights but could not reproduce effects in a subsequent experiment attempting to induce responses in live performance. It was theorized that the lack of repeated response may have been the result of not accounting for tyrosine (Tyr) when formulating the Phe test diets [65]. Similarly, other researchers have focused on Phe despite the fact that the interconnected relationship of Phe and Tyr has been known in poultry since the 1940’s [2, 66–68]. Sasse and Baker [69] determined that the Tyr requirement makes up 42.5% of the Phe plus Tyr requirement, which has been reported to be 112% of digestible lysine [70]. Unfortunately the studies evaluating the Phe plus Tyr requirements have focused on the first 2 weeks of the chick’s life and therefore have left a void in literature, leading to the generation of suggested amino acid requirements that remain constant for the entire growout period [71]. As poultry diets are continually refined and amino acid excesses are minimized, the levels of dietary Phe and Tyr may influence diet formulation not only due to concerns of reduced growth performance (Table 2) but also in broiler health as Phe has been found to reduce mortality in the face of mycotoxin poisoning [72].

## Histidine and the need for further research

As diets continue to be lowered in CP, research on histidine (His) responses and its minimum in least cost formulation warrant attention (Table 2). In 1926, Cox and Rose [73] created a purified diet, virtually devoid of His by precipitation of hydrolyzed casein, and fed the diet to rats with and without purine supplementation in a long-term study (100 plus days). Dietary addition of adenine, guanine, creatinine, creatine, and their combinations failed to improve performance in the His deficient diet indicting the essentiality of His via irreversible purine synthesis [73]. However, in 1959 Leveille and Fisher [74] assessed His needs for maintenance using a His-free diet in the adult rooster in a short-term study (5-day feeding period) and determined it to be non-essential as measured by nitrogen balance. Subsequent work utilized the former His-free diet in the same laboratory in 1960 [75], but fed the His-free diet to adult roosters for 14 days, and found decreased breast muscle anserine and carnosine levels, indicating the essentiality of His. Further, Fisher et al. [76] fed adult cockerels protein depleted diets for 7 weeks and found that wet breast muscle had decreased carnosine and increased His, from which they concluded that the high breast His arose from carnosine breakdown or the decrease in its synthesis.

Robbins et al. [77] fed chicks His graduations and assessed growth performance, plasma free His, and breast muscle carnosine. It was determined that maximized stores of muscle carnosine and adequate growth based on His adequacy were required before plasma free His levels accumulated. It was concluded that His evaluation through reduced or His-free diets should include a pre-experimental adjustment period in order to reduce both muscle carnosine and plasma His so as not to underestimate the His response [77].

Kai et al. [78] fed diets with deficient (67% of requirement), adequate (100% of requirement), and excess (200% of requirement) His to broilers from days 15 to 24 and found that His deficiency impaired growth and breast meat. These reductions in growth and breast meat yield were also accompanied by a complete depletion in breast muscle carnosine [78]. Moreover, recent research in our laboratory [79] demonstrated that when diets containing approximately 89% of the His requirement were fed to broilers from 15 to 35 days of age, no negative effects were observed on body weight gain, but breast meat and right thigh yield were significantly reduced. These data suggest that broilers will mobilize bound carnosine to maintain growth, sacrificing carcass part yields in the process. Thus, low CP diets marginal in His in grower and finisher diet phases may result in unexpected yield losses at processing. Furthermore, assessing His needs in broilers warrants depletion diets, similar to those implemented in mineral studies, as suggested by Robbins et al. [77].

## Glutamine and intestinal recovery from low protein diets

A better understanding of the bird’s intestinal homeostasis for amino acid needs and dietary form of amino acids in low CP diets necessitates research. Feeding reduced CP diets to broilers has shown a marked increase in diet AME values and amino acid digestibility coefficients, but upon further investigation these changes appear to be the result of diet composition, and not a result of the birds’ metabolism [11, 22, 80]. With feed-grade amino acids making up a larger portion of broiler diets, amino acids are able to bypass the gut and enter the blood stream without the need of digestion [25]. Unfortunately, this beneficial attribute of reduced CP diets may not be as beneficial as it seems when the status of the gut is examined. Various researchers have reported negative effects on live performance and intestinal morphology when feeding reduced CP diets [81–83]. These negative responses may be the result of how the gastrointestinal tract feeds through first pass metabolism. Increased feed grade amino acid usage is accompanied by a decrease in non-essential amino acids, adhering to the ideal protein concept, which deprives the gut mucosa of its primary energy source. When considering nonessential amino acids, the gut mucosa consumes and utilizes 66%, 98%, and 99% of Gln, glutamate, and aspartate, respectively, from intraluminal sources before they reach the blood supply [84]. Of these three, only Glu can be measured in arterial blood supply post absorption [84]. Non-essential amino acid reports in the literature are predominated by Gly, which, unlike Gln, is not used to the extent in the bird’s lumen [84]. As CP continues to be reduced, therefore, non-essential amino acid supply for intestinal integrity (e.g., Gln) warrants attention (Table 2).

Bortoluzzi et al. [85] reviewed Gln and indicated that its supplementation may reduce intestinal atrophy and aid in mucosal repair following trauma. The former review [85] focused on pathogen challenges, particularly coccidiosis, and hypothesized that amino acid supplementation could aid in supporting the bird’s intestinal integrity in the absence of subtherapeutic antibiotic use in diets. Under reduced CP conditions the trauma in which the gut is subjected to could be a form of starvation due to the reduced amount of available nonessential amino acids. The intestinal changes observed under reduced CP conditions include reduced villi height and absorptive surface area, sharing some commonalities with a coccidia infection [82, 83, 86]. Further, Gln supplementation has been shown to increase intestinal weight and villi height [87, 88], in addition to providing intraluminal and arterial energy [84]. Nontraditional levels of amino acids may be needed in reduced CP diets to offset the reduction in nonessential amino acid levels in order to maintain and stimulate gut development (Table 2). Further, nitrogen supply in the lumen and its effect on pathogenic bacteria is not entirely understood.

## Conclusions for linear programming

Realized feeding of reduced CP diets for broilers requires constant attention to the linear programming nutrient matrix. For example, formulators are considering establishing true protein values for ingredients to better reflect cereal and oilseed nitrogen levels [89] (Table 2). Chronological events of linear programing in the U.S. from the 1950’s to present has been reviewed [7], and include: removal of CP nutrient minimums, removal of feed grade amino acid ingredient maximum, addition of all amino acids on a digestible basis in the ingredient and nutrient matrix, and expression of digestible amino acids to Lys ratios in the formula matrix (Table 1). The former events became realized as feed grade amino acid prices decreased and resulted in lower diet costs. Although feed cost is the highest contributor to broiler production costs, today’s nutritionists are evaluating the reduction in nitrogen output while considering unique, and somewhat revolutionary, criteria (e.g., bird water intake, nutrient intake as a function of life-cycle analysis, welfare, and soil and air quality). For example, Table 2 is provided to summarize amino acid responses while considering both the ingredient and nutrient matrix in least cost formulation to provide commercial nutritionists information on reducing nitrogen excesses and researchers with concepts for improving nitrogen efficiency.

## Data Availability

Data may be provided following request to the corresponding author.

## Abbreviations

BCAA: Branched-chain amino acids
CP: Crude protein
Gln: Glutamine
Gly: Glycine
His: Histidine
Ile: Isoleucine
Leu: Leucine
Lys: Lysine
Met: Methionine
Phe: Phenylalanine
Ser: Serine
Thr: Threonine
Tyr: Tyrosine
Val: Valine
